# Ru(III) Complexes with Lonidamine-Modified Ligands

**DOI:** 10.3390/ijms222413468

**Published:** 2021-12-15

**Authors:** Ilya A. Shutkov, Yulia N. Okulova, Vladimir Yu. Tyurin, Elena V. Sokolova, Denis A. Babkov, Alexander A. Spasov, Yulia A. Gracheva, Claudia Schmidt, Kirill I. Kirsanov, Alexander A. Shtil, Olga M. Redkozubova, Elena F. Shevtsova, Elena R. Milaeva, Ingo Ott, Alexey A. Nazarov

**Affiliations:** 1 Department of Medicinal Chemistry & Fine Organic Synthesis, Lomonosov Moscow State University, 1/3 Leninskie Gory, 119991 Moscow, Russia; ilya-shutkov@med.chem.msu.ru (I.A.S.); nosovayulia@yahoo.com (Y.N.O.); olivecc@mail.ru (V.Y.T.); jullina74@mail.ru (Y.A.G.); milaeva@med.chem.msu.ru (E.R.M.); 2 Scientific Center for Innovative Drugs, Volgograd State Medical University, 39 Novorossiyskaya Street, 400087 Volgograd, Russia; sokolova210795@gmail.com (E.V.S.); denis.a.babkov@gmail.com (D.A.B.); aspasov@mail.ru (A.A.S.); 3 Institute of Medicinal and Pharmaceutical Chemistry, Technische Universität Braunschweig, 55 Beethovenstrasse, 38106 Braunschweig, Germany; cla.schmidt@tum.de (C.S.); ingo.ott@tu-braunschweig.de (I.O.); 4 Blokhin Cancer Research Center, 24 Kashirskoye Shosse, 115478 Moscow, Russia; kkirsanov85@yandex.ru (K.I.K.); shtilaa@yahoo.com (A.A.S.); 5 Institute of Medicine, RUDN University, 6 Miklukho-Maklaya St., 117198 Moscow, Russia; 6 Neurobotics, Zelenograd pas. 4922, 4-2-477, 124498 Moscow, Russia; redkozubova@gmail.com; 7 Institute of Physiologically Active Compounds, Russian Academy of Sciences, 1 Severniy Proezd, 142432 Chernogolovka, Russia; e.f.shevtsova@gmail.com

**Keywords:** lonidamine, redox balance, cell death, antiproliferative activity, thioredoxin reductase

## Abstract

A series of bifunctional Ru(III) complexes with lonidamine-modified ligands (lonidamine is a selective inhibitor of aerobic glycolysis in cancer cells) was described. Redox properties of Ru(III) complexes were characterized by cyclic voltammetry. An easy reduction suggested a perspective for these agents as their whole mechanism of action seems to be based on activation by metal atom reduction. New compounds demonstrated a more pronounced antiproliferative potency than the parental drug; individual new agents were more cytotoxic than cisplatin. Stability studies showed an increase in the stability of complexes along with the linker length. A similar trend was noted for antiproliferative activity, cellular uptake, apoptosis induction, and thioredoxin reductase inhibition. Finally, at concentrations that did not alter water solubility, the selected new complex evoked no acute toxicity in Balb/c mice.

## 1. Introduction

**Figure 1:**
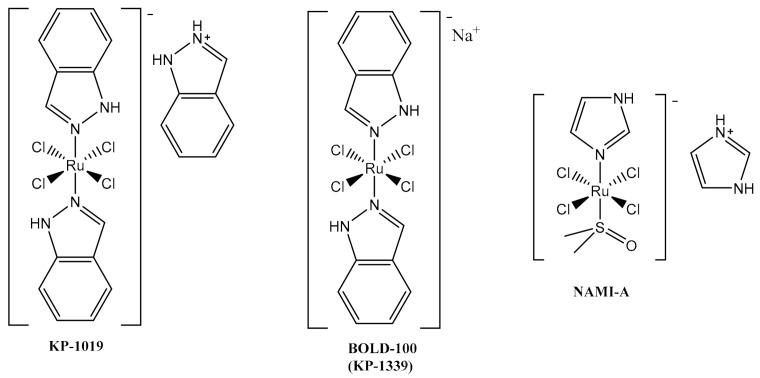
The structures of Ru(III) complexes in clinical trials.

Since the discovery of the antitumour activity of cisplatin, organic complexes with other metals such as ruthenium, gold, osmium, gallium, rhodium, titanium, etc., have been investigated. Ruthenium compounds became the most promising because of their different mode of action and relatively low general toxicity. The Ru(III) compounds KP1019 (eventually changed to the sodium salt known as KP1339; BOLD 100) and NAMI-A (Figure 1) became the first metal-based non-platinum drugs that entered clinical trials [1,2,3,4,5,6,7].

Compound NAMI-A exhibits an antimetastatic activity but is less active toward primary tumours [8], which has been attributed to a specific mode of action [9,10,11]. KP1339 was found to be active against drug-resistant cell lines [12,13,14,15].

**Figure 2:**
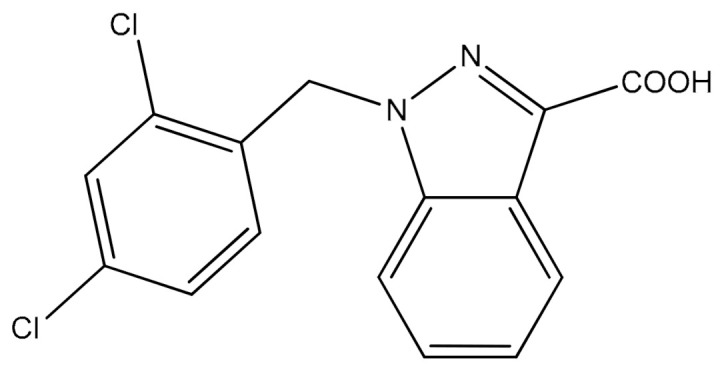
Structure of 1-(2,4-dichlorobenzyl)-1H-indazole-3-carboxylic acid (lonidamine).

One significant metabolic characteristic of malignant cells is their activated glycolysis [16,17]. Hexokinase II (HKII) catalyses the first stage of aerobic glycolysis, thereby inducing glycolysis and limiting its rate [18,19]. Lonidamine (Figure 2) is an inhibitor of mitochondrial hexokinase. This agent stimulates lactate production in normal cells and inhibits glycolysis in malignant counterparts [20,21]. Lonidamine is widely investigated in clinical trials for the treatment of different types of cancer [22,23,24] and has recently been recognised as a drug candidate for COVID-19 patients [25], alongside some metal-based compounds [26].

Modifying the known metal-based drugs by introducing a biologically active molecule is a promising approach in medicinal chemistry to improve cytotoxicity, selectivity, and the twin-drug effect [27,28,29,30]. Recently, we introduced lonidamine in Pt(IV) or Ru(II) moieties and obtained compounds with increased activity and selectivity [31,32,33,34]. Pt(IV) compounds showed a significant increase of antiproliferative activity superior to cisplatin and lonidamine [32,35], and Ru(II) complexes were specifically active against glioblastoma cell lines [31]. Thioredoxin reductases (TrxR) belong to the thioredoxin system along with NADPH and thioredoxin (Trx). TrxR enzymes are overexpressed in cancer cells, ensuring the resistance of their phenotype to high ROS levels [36,37]. Thus, TrxR is a target for developing new metal-based anticancer agents, including ruthenium and gold complexes [37,38,39,40].

In this study, we describe the synthesis of Ru(III) complexes with lonidamine-modified imidazole ligands and report their cytotoxicity, electrochemical behaviour, stability, lipophilicity, intracellular accumulation, as well as mechanisms of cell death and in vivo tolerance.

## 2. Results and Discussion

### 2.1. Synthesis

**Scheme 1:**
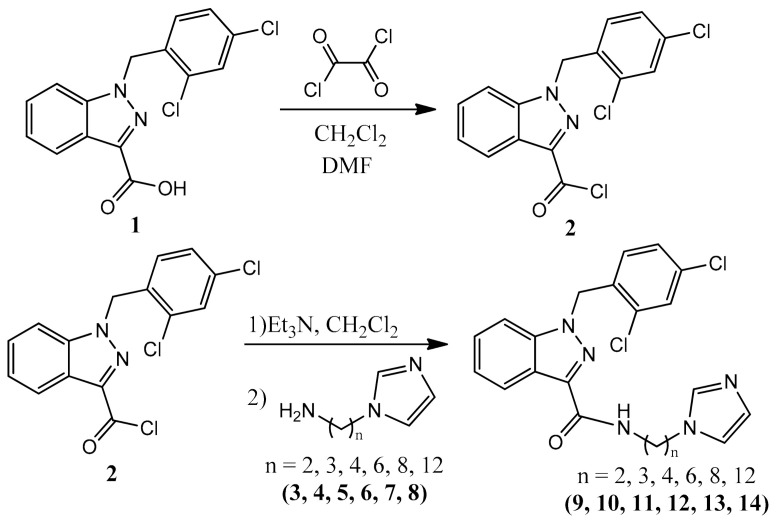
Synthesis of ligands.

Ligands (**9**–**14**) were obtained by the reaction of 1-(2,4-dichlorobenzyl)-1*H*-indazole-3-carbonyl chloride (**2**) with corresponding imidazolamines (**3**–**8**) in CH_2_Cl_2_ using an excess of amine or triethylamine as an HCl acceptor (Scheme 1). Products were isolated by column chromatography on silica gel and characterised by both NMR spectroscopy (^1^H and ^13^C{^1^H}) and elemental analyses.

**Scheme 2:**
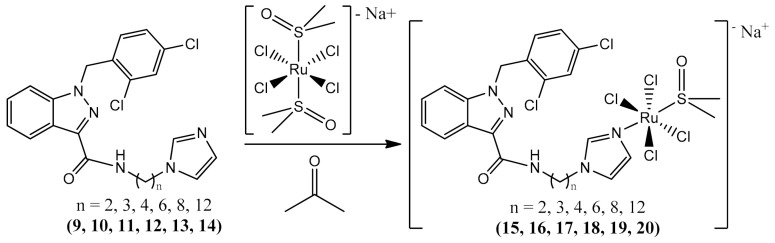
Synthesis of Ru(III) complexes.

Ru(III) complexes (**15**–**20**), the analogues of NAMI complexes, were prepared as described [41] by substituting DMSO in Na[Ru(DMSO)_2_] with the imidazole moiety of new ligands (Scheme 2).

**Figure 3:**
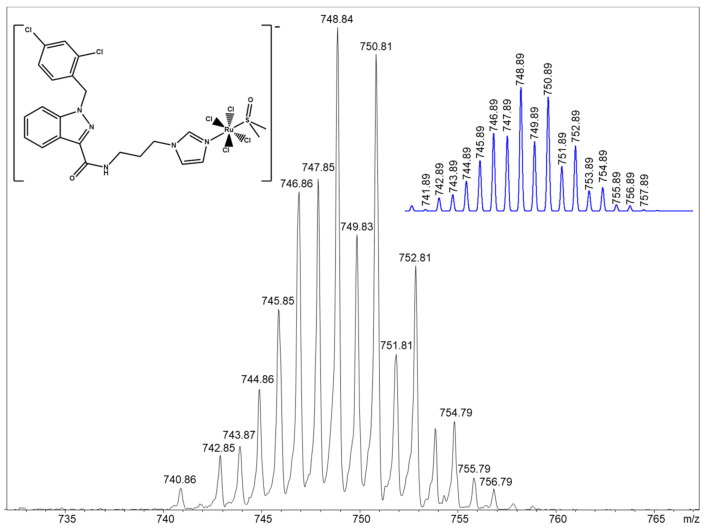
ESI mass spectrum of complex **16**.

Only complex **16** precipitated from the reaction mixture after 5 h; **16** was isolated by filtration. Other complexes were isolated by flash column chromatography on silica gel (eluent: acetone) after stirring the reaction mixture for 10 h. Formation of the desired complexes was proved by ESI mass spectrometry; purity was confirmed by elemental analysis. The most abundant peak in the ESI mass spectra of complexes was assigned as [M–Na^+^]^−^ and the isotopic distribution was in good agreement with the calculated values (Figure 3).

The stability of Ru(III) complexes (**15**–**20**) was investigated in a solution resembling physiological conditions (20 mM of phosphate buffer, pH 7.4, and 100 mM of NaCl at 37 °C) by UV-vis spectrophotometry (Table 1). The half-transformation time t_1/2_ was calculated based on changes in the electronic absorption spectrum (Figure S1) and defined as the value of t at the A_lin_/2 point. ΔA(t) was plotted against λ_max_; the initial section was approximated as a linear function. We found that the stability of complexes increased along with the length of the alkyl linker. Complex **20** was the most stable (t_1/2_ ~ 35 min), whereas **15**–**17** were unstable (t_1/2_ ~ 5 min).

The lipophilicity of complexes **15**–**20** was studied using a standard shake-flask method in a mixture of water/n-octanol. For the quantification of compounds in the water phase, UV-vis spectrophotometry was used. Fast hydrolysis of **15**–**17** complicated the measurements, therefore we failed to obtain the results. Among other complexes, as expected, **20** was the most lipophilic (Table 1).

### 2.2. Electrochemical Studies of Ru(III) Complexes

The Ru(III) complexes are kinetically inert compared to the Ru(II) counterparts. It is assumed that the mechanism of action of Ru(III) complexes includes a reduction to Ru(II) compounds, which are more labile in substitution reactions and react with specific regions of proteins [12,42]. Reduction of complexes under physiological conditions occurs in the presence of glutathione, ascorbic acid, and cysteine. To be activated by reduction, the Ru(III) complexes should have biologically attainable reduction potentials (approximately −0.4–0.9 V with respect to the Ag/AgCl reference electrode).

The redox behaviour of ligand **10** and complexes **15**–**20** was studied on Pt and glassy carbon (GC) electrodes in CH_3_CN and CH_2_Cl_2_. In the case of the ligand, redox transitions were not observed at any applied potentials, up to the discharge of the n-Bu_4_NBF_4_ background electrolyte. At the same time, complexes **15**–**20** were electroactive and exhibited two redox processes with respect to the metal centres in the range of +2 V to −2 V.

**Figure 4:**
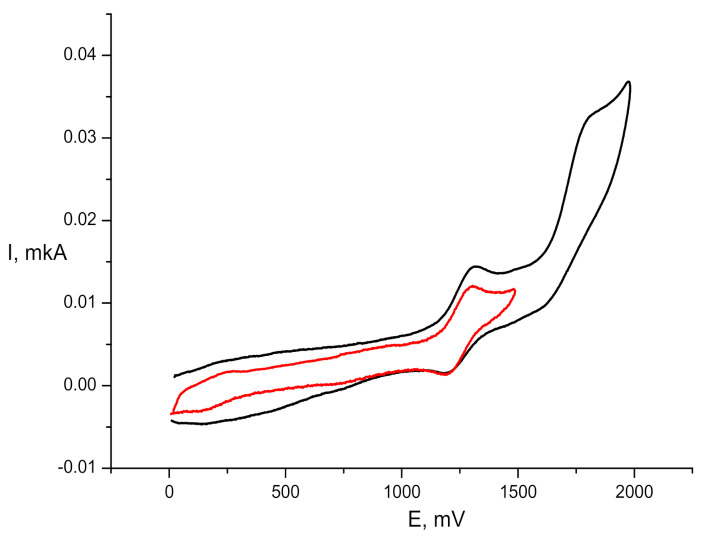
The cyclic voltammogram of complex **16** in the anodic range (sweep rate 100 mV/s, C = 1 × 10^−3^ M, n-Bu_4_NBF_4_, and rel. Ag|AgCl|KCl(sat.)).

**Figure 5:**
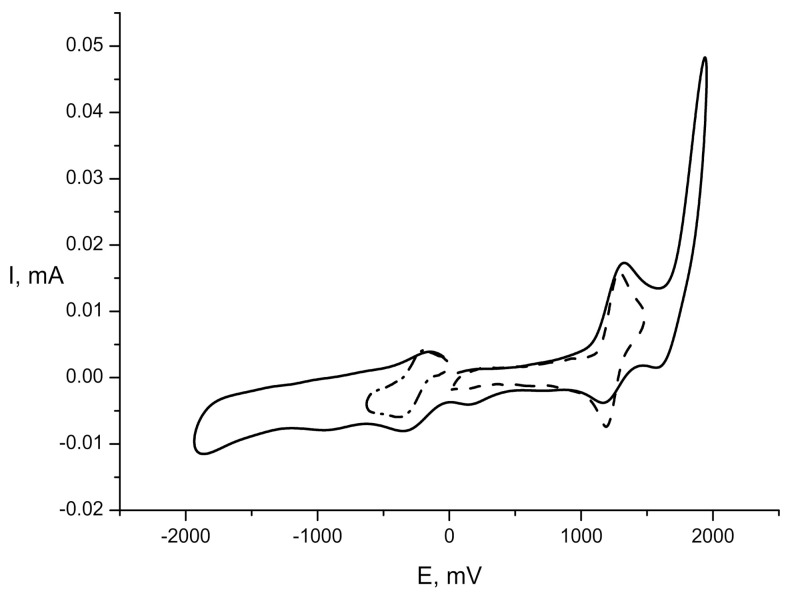
Cyclic voltammogram of complex **16** on the GC electrode (sweep rate 100 mV/s, C = 1 × 10^−3^ M, n-Bu4NBF4, and rel. Ag|AgCl|KCl(sat.)).

Potentials of redox transitions are summarised in Table 2 and Table 3; representative voltammograms are shown in Figure 4 and Figure 5. In the anodic region, voltammograms of complexes demonstrated two oxidative responses. In both CH_3_CN and CH_2_Cl_2_ using as a solvent, a one-electron reversible peak was recorded in the 1.19–1.3 V range corresponding to the oxidation of Ru (III) to Ru (IV; Figure 4 and Figure 5). The two-electron quasi-reversible peak appeared at more positive potentials, namely 1.60–1.97 V, in the case of CH_3_CN (Table 2), which has not been described for Ru complexes. Explanation of the nature of the second peak requires more investigation because such peaks were not observed on voltammograms of ligands.

Moreover, in the anode region of CVA in CH_2_Cl_2_, compounds **15**–**19** showed quasi-reversible peaks of low intensity in the 0.15–0.58 V region (Table 3). According to the literature [43], this peak can be due to the redox transition of Ru(II) into Ru(III) with a changed ligand environment.

In the negative potential range of complexes **15**–**20**, a one-electron peak was observed at values from −80 to −310 mV on the cathodic scan when measured on the Pt electrode and at −128 to −410 mV on the GC electrode in CH_3_CN. These values correspond to the process of the reduction of Ru (III) in Ru (II) [41,42,44,45]. The peak of Ru(II)→Ru(III) oxidation appeared during the reverse scan of the potential (Figure 5).

The quasi-reversible nature of the peaks (the difference between cathodic and anodic potentials is ΔE = 150–260 mV on direct and reverse scans) points to the changed geometry of the complexes. Based on CVs, one may conclude that the length of the hydrocarbon linker in the ligand insignificantly affects the values of redox potentials. The redox behaviour of complexes also weakly depended on the nature of the working electrode and the solvent, although in the case of CH_2_Cl_2_, the Ru(III)/Ru (II) reduction was observed at bigger negative potentials (Table 3). An easy reduction of complexes **15**–**20** indicates the promise of their use as antitumour compounds whose efficacy is based on metal atom reduction.

### 2.3. Cellular Ruthenium Accumulation 

**Figure 6:**
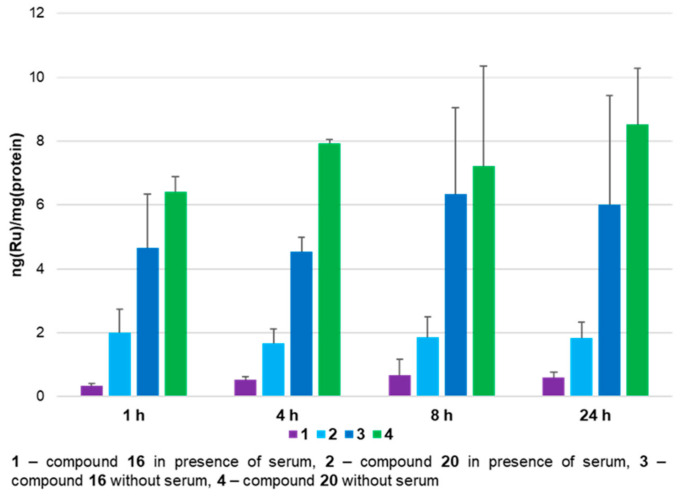
Ruthenium content in MCF7 cells.

The cellular accumulation of ruthenium complexes was studied by atomic absorption spectrometry. The MCF-7 breast carcinoma cells were incubated with **16** and **20** for 1–24 h in DMEM with or without fetal bovine serum to reduce the binding of complexes with proteins. The accumulation was dependent on the linker’s length. Complex **20** (n = 12) accumulated more readily than **16** (n = 3). The ruthenium complex entered the cells relatively fast; by 4 h, no notable further increase of the Ru content was observed (Figure 6). Notably, the cellular Ru accumulation was affected by the presence of serum in the cell culture media. In particular with complex **16**, only very low uptake was observed when serum was present in the culture medium. For both complexes **16** and **20**, significantly higher cellular ruthenium concentrations were determined when the experiments were done with serum-free cell culture medium. The negative effect of serum on the cellular Ru accumulation has been reported for other ruthenium species; however, significant effects were detectable only after longer exposure (24 h) [46].

### 2.4. Inhibition of TrxR

**Figure 7:**
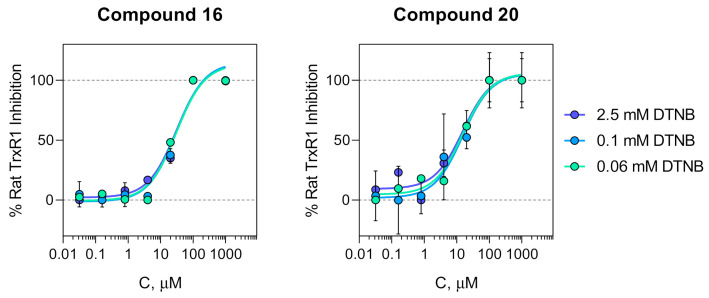
TrxR1 inhibition in rat liver extracts.

Complexes **16** and **20** inhibited the activity of isolated rat TrxR1 at micromolar concentrations: IC_50_ (**16**) = 20.4 ± 0.1 µM and IC_50_ (**20**) = 8.5 ± 0.3 µM. We demonstrated the efficacy of **16** and **20** as modulators of intracellular TrxR1 using rat liver extracts and varying concentrations of each complex (Figure 7 and Table S1; see Experimental for details) [47]. Both compounds were micromolar TrxR1 inhibitors, with compound **20** being ca. two-folds more potent compared to **16**. The efficacy of each compound was independent of the DTNB concentration (*p* = 0.75 for **16**; *p* = 0.99 for **20**), which indicates a non-competitive mechanism of inhibition. This is in line with previously reported metal-based non-competitive TrxR1 inhibitors (gold [48] and gadolinium [49] compounds or lanthanum chloride [50]). Inhibition constants were estimated according to the Cheng–Prusoff equation as *K*_i_ = 30.7 µM for **16** and *K*_i_ = 15.3 µM for **20**.

### 2.5. Cell Death Studies

The antiproliferative activity of new ligands and complexes was determined against human cell lines, including colon adenocarcinoma SW480, breast adenocarcinoma MCF-7, lung adenocarcinoma A549, neuroblastoma SHSY5Y, and non-tumourigenic HaCaT (Table 4). In general, complexes were more cytotoxic compared to ligands, lonidamine, and, in some cases, cisplatin. Complex **20** was the most potent. We chose this compound and the synthetically accessible complex **16** for mechanistic studies.

**Figure 8:**
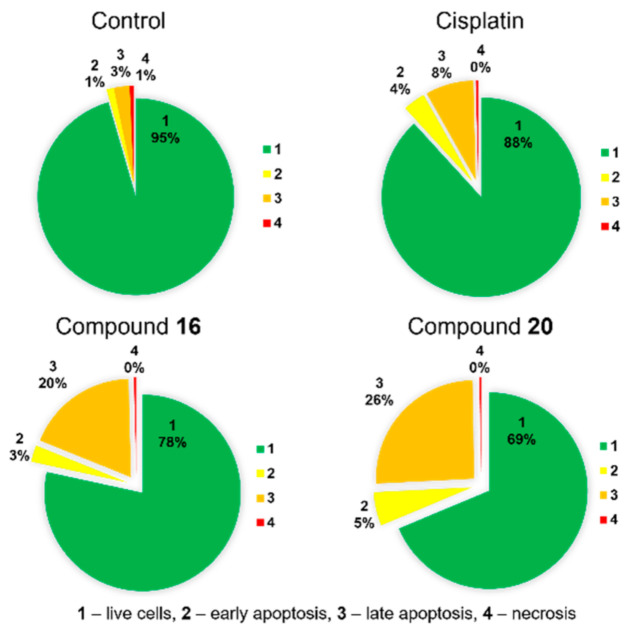
Parameters of cell death detectable by flow cytometry in HCT116 cells treated with **16**, **20,** and cisplatin. Shown is one representative experiment out of four with similar results.

Mechanisms of cell death, that is, Annexin V/7-AAD reactivity and caspase 3/7 activation [51], were determined for **16** and **20** in HCT116 cells using the Muse^®^ Annexin V & Dead Cell Kit (Luminex corp., Austin, TX, USA) and flow cytometry. Cells were incubated with **16**, **20,** or cisplatin (reference drug) for 24 h at concentrations corresponding to 2xIC_50_ values (obtained in MTT assays; Table 4). The percentages of Annexin V-positive cells in response to **16** and **20** were higher than cisplatin (Figure 8). Complex **20** with the C12 linker induced Annexin V positivity slightly more efficiently than **16** with the C3 linker (27.4 ± 2.1% vs. 19.7 ± 0.6%).

**Figure 9:**
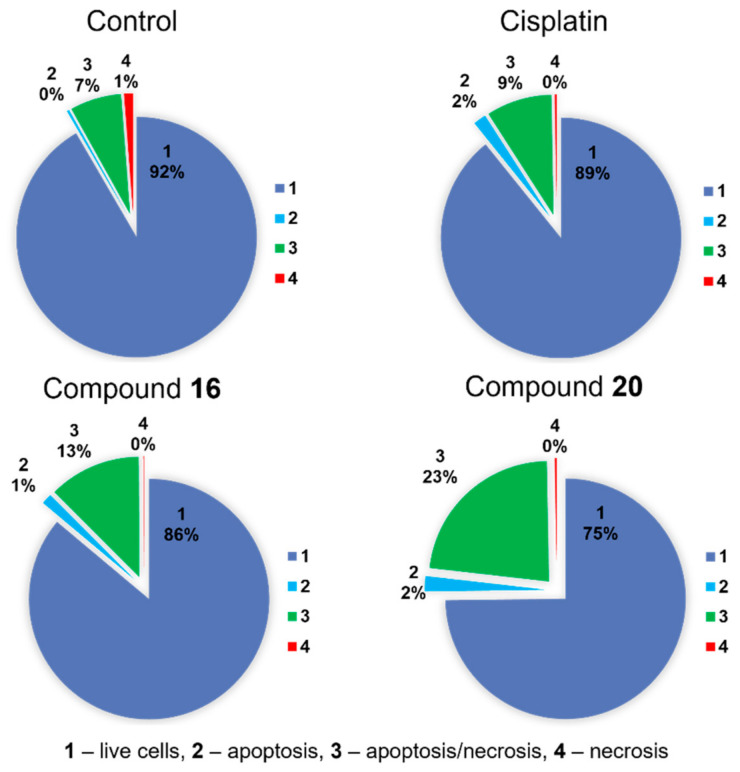
Cytometry studies of caspase 3/7 activation on the HCT116 cell line.

Caspase activation was studied using the Muse^®^ Caspase-3/7 Kit (Luminex corp., Austin, TX, USA). Similarly to Annexin V reactivity (Figure 9), complexes activated caspases 3/7 processing more potently than cisplatin. Complex **20** with the longer linker was the most active. 

To visualise the activity of complex **20** in caspase activation, the fluorescent kit CellEvent™ Caspase-3/7 Green ReadyProbes™ Reagent was applied. After incubation of cells with **20,** the kit reagent was added and the formation of bright green fluorescent cells with activated caspases was observed (Figure S2).

### 2.6. Tolerance of ***16*** In Vivo

Finally, we tested the acute toxicity of complex **16** in Balb/c mice after a single bolus i.p. injection of the compound dissolved in saline. The range of doses was 70–110 mg/kg; higher doses were not achievable due to limited water solubility. Animals were monitored for 21 days after injection. As shown in Table 5, no deaths were detected after injections of 70 mg/kg or 80 mg/kg. Mice in these cohorts had normal hair cover; no changes of nutritional behaviour were registered over the entire period of observation. In contrast, doses >80 mg/kg were lethal for individual animals. Tremor and dyspnoea were registered within the initial 1–2 h after injection of 110 mg/kg. These manifestations gradually subsided; however, mice became less active. Deaths were registered over the next 1–2 days (Table 5). In each group, the survived animals remained without visible changes for up to 21 days after the injection of **16**.

## 3. Materials and Methods

Reagents were purchased from Aldrich unless specified otherwise. All solvents were purified and degassed prior to use. NMR spectra were recorded on a Bruker FT-NMR Avance III 500 MHz instrument at 500.32 (^1^H), 125.81 (^13^C) MHz. Two-dimensional NMR measurements were carried out using standard pulse programs. Chemical shifts were referenced relative to the solvent signal for ^1^H and ^13^C spectra. ESI mass spectra were recorded on a LC/MSn ion trap mass spectrometer amaZon SL (Bruker, Bremen, Germany) with MeOH as a solvent. Elemental analysis was performed at Moscow State University with the MicroCube Elementar analyser. Melting points were determined with a Stuart Scientific SMP3 apparatus and uncorrected. UV-vis spectra were recorded on Thermo Scientific Evolution 300. Source of cell lines: initially, all cell lines were purchased from ATCC (Manassas, VA, USA) and routinely propagated by the authors according to the manufacturer’s protocols. 

### 3.1. N-(2-(1H-Imidazol-1-yl)ethyl)-1-(2,4-dichlorobenzyl)-1H-indazole-3-carboxamide *(**9**)*

Oxalyl chloride (4.5 mL, 0.0525 mol) and a catalytic amount of DMF were added to the solution of lonidamine (893 mg, 2.78 mmol) in DCM (50.0 mL). The reaction mixture was refluxed for 1 h. The solvent and oxalyl chloride were removed in a vacuum. The obtained chloroanhydride without purification was dissolved in DCM (50.0 mL) and triethylamine (774 μL, 5.56 mmol), and then **3** (300 mg, 2.70 mmol) was added with stirring. The reaction mixture was stirred for 8 h, then washed by NaHCO_3_ solution (2 × 100 mL) and NaCl solution (2 × 100 mL), and dried on Na_2_SO_4_. The solvent was removed and the product was purified by column chromatography on silica gel (eluent: CH_2_Cl_2_:EtOH 12:1, R_f_ = 0.4).Yield: 387 mg (34.6%), m.p. 120–121 °C, elem. anal. calc. (%) for C_20_H_17_Cl_2_N_5_O: C 57.98, H 4.14, and N 16.90. Found: C 58.19, H 4.14, and N 16.58. **^1^H NMR** (400.13 MHz, CDCl_3_) δ: 8.39 (d, 1H, J = 8.2 Hz, H4), 7.53 (s, 1H, H20), 7.48–7.30 (m, 4H, H5_,_ H6, H7, H12), 7.19 (t, 1H, J = 5.7 Hz, NH), 7.13 (d, 1H, J = 8.4 Hz, H14), 7.10 (s, 1H, H19), 6.99 (s, 1H, H18), 6.69 (s, 1H, J = 8.3 Hz, H15), 5.65 (s, 2H, H9), 4.26 (t, 2H, J = 5.8 Hz, H17), and 3.82 (q, 2H, J = 5.9 Hz, H16). **^13^C{^1^H}NMR**(100.61 MHz, CDCl_3_) δ: 162.8(C1), 141.2(C2), 137.7(C8), 137.4(C20), 134.6(C10), 133.3(C11/C13), 132.1(C15), 129.9(C19), 129.5(C12), 127.6(C14), 127.5(C6), 123.3(C4), 122.9(C3), 122.8(C5), 119.0(C18), 109.4(C7), 50.1(C9), 46.5(C17), and 40.3(C16).


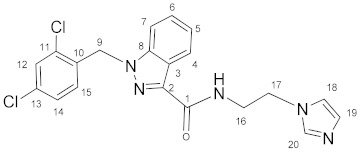


### 3.2. N-(3-(1H-Imidazol-1-yl)propyl)-1-(2,4-dichlorobenzyl)-1H-indazole-3-carboxamide *(**10**)*

Oxalyl chloride (1.6 mL, 0.0187 mol) and a catalytic amount of DMF were added to the solution of lonidamine (300 mg, 0.94 mmol) in DCM (16.0 mL). The reaction mixture was refluxed for 1 h. The solvent and oxalyl chloride were removed in vacuum. The obtained chloroanhydride without purification was dissolved in DCM (16.0 mL) and triethylamine (261 μL, 1.88 mmol), and then **4** (114 mg, 0.91 mmol) was added with stirring. The reaction mixture was stirred for 8 h, then washed by NaHCO_3_ solution (2 × 30 mL) and NaCl solution (2 × 30 mL), and dried on Na_2_SO_4_. The solvent was removed and the product was purified by column chromatography on silica gel (eluent: CH_2_Cl_2_:EtOH 12:1, R_f_ = 0.4).Yield: 270 mg (69.2%), m.p. 121–122 °C, elem. anal. calc. (%) for C_21_H_19_Cl_2_N_5_O: C 58.89, H 4.47, and N 16.35. Found: C 58.55, H 4.57, and N 16.06. **^1^H NMR** (400.13 MHz, CDCl_3_) δ: 8.38 (d, 1H, J = 7.9 Hz, H4), 7.51 (s, 1H, H20), 7.40–7.27 (m, 4H, H5, H6, H7, H12), 7.17–7.05 (m, 2H, H14, NH), 7.03 (s, 1H, H19), 6.95 (s, 1H, H18), 6.62 (d, 1H, J = 8.5 Hz, H15), 5.61 (s, 2H, H9), 4.04 (t, 2H, J = 7.0 Hz, H17), 3.50–3.45 (m, 2H, H16), and 2.15–2.08 (m, 2H, H21). **^13^C{^1^H}** **NMR** (100.61 MHz, CDCl_3_) δ: 162.8(C1), 141.2(C2), 138.1(C8), 137.1(C20), 134.5(C10), 133.2(C11, C13), 132.3(C15), 129.6(C19), 129.5(C12), 129.3(C14), 127.6(C4/C6), 127.5(C4/C6), 123.1(C3/C5), 122.9(C3/C5), 118.9(C18), 109.3(C7), 50.0(C9), 44.6(C17), 36.2(C16), and 31.5(C21).


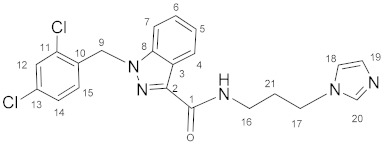


### 3.3. N-(4-(1H-Imidazol-1-yl)butyl)-1-(2,4-dichlorobenzyl)-1H-indazole-3-carboxamide *(**11**)*

Oxalyl chloride (4.5 mL, 0.0525 mol) and a catalytic amount of DMF were added to the solution of lonidamine (829 mg, 2.58 mmol) in DCM (40.0 mL). The reaction mixture was refluxed for 1 h. The solvent and oxalyl chloride were removed in vacuum. The obtained chloroanhydride without purification was dissolved in DCM (40.0 mL) and triethylamine (718 μL, 5.16 mmol), and then **5** (348 mg, 2.50 mmol) was added with stirring. The reaction mixture was stirred for 8 h, then washed by NaHCO_3_ solution (2 × 80 mL) and NaCl solution (2 × 80 mL), and dried on Na_2_SO_4_. The solvent was removed and the product was purified by column chromatography on silica gel (eluent: CH_2_Cl_2_:EtOH 12:1, R_f_ = 0.4).Yield: 841 mg (76.1%), m.p. 98–100 °C, elem. anal. calc. (%) for C_22_H_21_Cl_2_N_5_O: C 59.74, H 4.79, and N 15.83. Found: C 60.13, H 4.91, and N 15.66. **^1^H NMR** (400.13 MHz, CDCl_3_) δ: 8.36 (d, 1H, J = 8.0 Hz, H4), 7.61 (s, 1H, H20), 7.43–7.22 (m, 4H, H5, H6, H7, H12), 7.14–6.99 (m, 3H, H14, H19, NH), 6.90 (s, 1H, H18), 6.59 (d, 1H, J = 8.3 Hz, H15), 5.60 (s, 2H, H9), 3.99 (t, 2H, J = 6.9 Hz, H17), 3.47 (q, 2H, J = 6.3 Hz, H16), 1.93–1.79 (m, 2H, H21/H22), and 1.66–1.54 (m, 2H, H21/H22). **^13^C{^1^H}** **NMR** (100.61 MHz, CDCl_3_) δ: 162.5(C1), 141.1(C2), 138.2(C8), 136.9(C20), 134.4(C10), 133.1(C11, C13), 132.3(C15), 129.4(C12/C19), 129.3(C12/C19), 128.7(C14), 127.6(C6), 127.4(C4), 123.0(C3/C5), 122.9(C3/C5), 118.9(C18), 109.2(C7), 50.0(C9), 46.7(C17), 38.0(C16), 28.3(C21/C22), and 26.9(C21/C22).


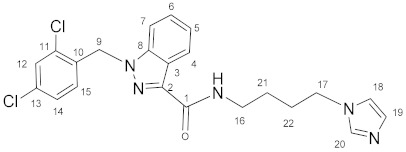


### 3.4. N-(6-(1H-Imidazol-1-yl)hexyl)-1-(2,4-dichlorobenzyl)-1H-indazole-3-carboxamide *(**12**)*

Oxalyl chloride (4.0 mL, 0.0466 mol) and a catalytic amount of DMF were added to the solution of lonidamine (700 mg, 2.18 mmol) in DCM (40.0 mL). The reaction mixture was refluxed for 1 h. The solvent and oxalyl chloride were removed in vacuum. The obtained chloroanhydride without purification was dissolved in DCM (40.0 mL) and triethylamine (607 μL, 4.36 mmol), and then **6** (355 mg, 2.12 mmol) was added with stirring. The reaction mixture was stirred for 8 h, then washed by NaHCO_3_ solution (2 × 80 mL) and NaCl solution (2 × 80 mL), and dried on Na_2_SO_4_. The solvent was removed and the product was purified by column chromatography on silica gel (eluent: CH_2_Cl_2_:EtOH 12:1, R_f_ = 0.4).Yield: 247 mg (24.8%), elem. anal. calc. (%) for C_24_H_25_Cl_2_N_5_O*0.1CH_2_Cl_2_: C 60.44, H 5.30, and N 14.62. Found: C 60.48, H 5.09, and N 14.32. **^1^H NMR** (400.13 MHz, CDCl_3_) δ: 8.43 (d, 1H, J = 8.2 Hz, H4), 7.50–7.27 (m, 5H, H5, H6, H7, H12, H20), 7.14–7.01 (m, 3H, H14, H19, NH), 6.90 (s, 1H, H18), 6.62 (d, 1H, J = 8.4 Hz, H15), 5.66 (s, 2H, H9), 3.93 (t, 2H, J = 7.1 Hz, H17), 3.49 (q, 2H, J = 6.9 Hz, H16), 1.87–1.74 (m, 2H, H21/H24), 1.72–1.59 (m, 2H, H21/H24), and 1.52–1.30 (m, 4H, H22, H23). **^13^C{^1^H}** **NMR** (100.61 MHz, CDCl_3_) δ: 162.4(C1), 141.2(C2), 138.5(C8), 137.0(C20), 134.5(C10), 133.2(C11, C13), 132.4(C15), 129.5(C19/C12), 129.4(C19/C12), 129.3(C14), 127.6(C6), 127.4(C4), 123.1(C3/C5), 123.0(C3/C5), 118.7(C18), 109.2(C7), 50.0(C9), 46.9(C17), 38.8(C16), 31.0(C21–C24), 29.7(C21–C24), 26.4(C21–C24), and 26.3(C21–C24).


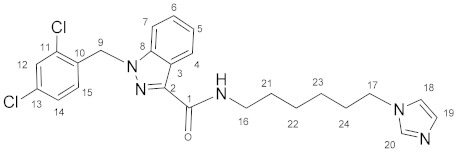


### 3.5. N-(8-(1H-Imidazol-1-yl)octyl)-1-(2,4-dichlorobenzyl)-1H-indazole-3-carboxamide *(**13**)*

Oxalyl chloride (3.5 mL, 0.0408 mol) and a catalytic amount of DMF were added to the solution of lonidamine (678 mg, 2.11 mmol) in DCM (35.0 mL). The reaction mixture was refluxed for 1 h. The solvent and oxalyl chloride were removed in vacuum. The obtained chloroanhydride without purification was dissolved in DCM (35.0 mL) and triethylamine (587 μL, 4.22 mmol), and then **7** (400 mg, 2.05 mmol) was added with stirring. The reaction mixture was stirred for 8 h, then washed by NaHCO_3_ solution (2 × 70 mL) and NaCl solution (2 × 70 mL), and dried on Na_2_SO_4_. The solvent was removed and the product was purified by column chromatography on silica gel (eluent: CH_2_Cl_2_:EtOH 12:1, R_f_ = 0.4).Yield: 419 mg (41.0%), elem. anal. calc. (%) for C_26_H_29_Cl_2_N_5_O*0.2CH_2_Cl_2_: C 61.05, H 5.75, and N 13.59. Found: C 61.22, H 5.49, and N 13.23. **^1^H NMR** (400.13 MHz, CDCl_3_) δ: 8.44 (d, 1H, J = 8.1 Hz, H4), 7.49–7.27 (m, 5H, H5, H6, H7, H12, H20), 7.14–7.00 (m, 3H, H14, H19, NH), 6.90 (s, 1H, H18), 6.61 (d, 1H, J = 8.4 Hz, H15), 5.66 (s, 2H, H9), 3.91 (t, 2H, J = 7.1 Hz, H17), 3.48 (q, 2H, J=6.8 Hz, H16), 1.82–1.71 (m, 2H, H21/H26), 1.70–1.59 (m, 2H, H21/H26), and 1.45–1.25 (m, 8H, H22-H25). **^13^C{^1^H}** **NMR** (100.61 MHz, CDCl_3_) δ: 162.3(C1), 141.2(C2), 138.6(C8), 137.0(C20), 134.4(C10), 133.1(C11, C13), 132.4(C15), 129.4(C12/C14/C18/C19), 129.3(C12/C14/C19), 129.3(C12/C14/C19), 127.6(C6), 127.4(C4), 123.2(C3/C5), 122.9(C3/C5), 118.7(C18), 109.2(C7), 50.0(C9), 47.0(C17), 39.0(C16), 31.0(C21–C26), 29.8(C21–C26), 29.0(C21–C26), 28.9(C21–C26), 26.8(C21–C26), and 26.5(C21–C26).


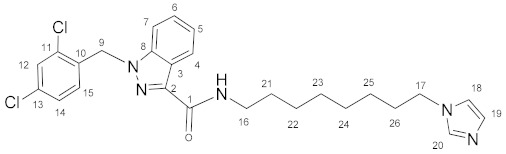


### 3.6. N-(12-(1H-Imidazol-1-yl)dodecyl)-1-(2,4-dichlorobenzyl)-1H-indazole-3-carboxamide *(**14**)*

Oxalyl chloride (1.8 mL, 0.0210 mol) and a catalytic amount of DMF were added to the solution of lonidamine (396 mg, 1.24 mmol) in DCM (20.0 mL). The reaction mixture was refluxed for 1 h. The solvent and oxalyl chloride were removed in vacuum. The obtained chloroanhydride without purification was dissolved in DCM (20.0 mL) and triethylamine (345 μL, 2.48 mmol), and then **8** (302 mg, 1.20 mmol) was added with stirring. The reaction mixture was stirred for 8 h, then washed by NaHCO_3_ solution (2 × 40 mL) and NaCl solution (2 × 40 mL), and dried on Na_2_SO_4_. The solvent was removed and the product was purified by column chromatography on silica gel (eluent: acetone, R_f_ = 0.4). Yield: 665 mg (47.9%), m.p. 86–88 °C, elem. anal. calc. (%) for C_30_H_37_Cl_2_N_5_O: C 64.97, H 6.73, and N 12.63. Found: C 65.02, H 6.72, and N 12.85. **^1^H NMR** (400.13 MHz, CDCl_3_) δ: 8.45 (d, 1H, J = 8.2 Hz, H4), 7.50–7.30 (m, 5H, H5, H6, H7, H12, H20), 7.12 (dd, 1H, J = 8.3, 2.1 Hz, H14), 7.07 (s, 1H, H19), 7.03 (t, 1H, J = 5.6 Hz, NH), 6.92 (s, 1H, H18), 6.62 (d, 1H, J = 8.3 Hz, H15), 5.69 (s, 2H, H9), 3.94 (t, 2H, J = 7.1 Hz, H17), 3.50 (q, 2H, J = 6.9 Hz, H16), 1.82–1.73 (m, 2H, H21/H30), 1.71–1.62 (m, 2H, H21/H30), and 1.46–1.23 (m, 16H, H22–H29). **^13^C{^1^H}** **NMR** (100.61 MHz, CDCl_3_) δ: 162.3(C1), 141.2(C2), 138.6(C8), 137.1(C20), 134.4(C10), 133.1(C11, C13), 132.5(C15), 129.5(C12/C14/C19), 129.4(C12/C14/C19), 129.2(C12/C14/C18/C19), 127.6(C6), 127.4(C4), 123.2(C3/C5), 123.0(C3/C5), 118.8(C18), 109.2(C7), 50.0(C9), 47.0(C17), 39.1(C16), 31.1(C21–C30), 29.8(C21–C30), 29.5(C21–C30), 29.4(C21–C30), 29.3(C21–C30), 29.1(C21–C30), 27.0(C21–C30), and 26.5(C21–C30).


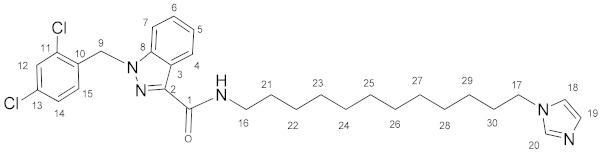


### 3.7. Na[trans-Ru(DMSO)(C_20_H_17_Cl_2_N_5_O)Cl_4_] *(**15**)*

Compound **9** (100 mg, 0.24 mmol) in ~3 mL of acetone was added to the solution of [Ru(DMSO)_2_Cl_4_]^−^Na^+^ (102 mg, 0.24 mmol) in acetone (40.0 mL). The reaction mixture was stirred for 10 h. The solution was filtered and evaporated up to a minimum volume. The product was purified by column chromatography on silica gel (eluent: acetone, R_f_ = 0.5). Yield: 107 mg (58.5%), m.p. 181–183 °C (decomp.), elem. anal. calc. (%) for C_22_H_23_Cl_6_N_5_NaO_2_RuS: C 34.85, H 3.06, N 9.24, and S 4.23. Found: C 35.07, H 3.38, N 8.99, and S 4.36, ESI-MS: *m*/*z*: 737 [M–Na^+^]^−^.

### 3.8. Na[trans-Ru(DMSO)(C_21_H_19_Cl_2_N_5_O)Cl_4_] *(**16**)*

Compound **10** (100 mg, 0.23 mmol) in ~3 mL of acetone was added to the solution of [Ru(DMSO)_2_Cl_4_]^−^Na^+^ (98 mg, 0.23 mmol) in acetone (40.0 mL). The reaction mixture was stirred for 10 h. After 5 h, a precipitate was formed. The solution was filtered and the product was washed by acetone and dried. Yield: 125 mg (69.4%), m.p. 203–206 °C (decomp.), elem. anal. calc. (%) for C_23_H_25_Cl_6_N_5_NaO_2_RuS: C 35.77, H 3.26, N 9.07, and S 4.15. Found: C 35.60, H 3.65, N 8.97, and S 3.90, ESI-MS: *m*/*z*: 749 [M–Na^+^]^−^.

### 3.9. Na[trans-Ru(DMSO)(C_22_H_21_Cl_2_N_5_O)Cl_4_] *(**17**)*

Compound **11** (100 mg, 0.23 mmol) in ~3 mL of acetone was added to the solution of [Ru(DMSO)_2_Cl_4_]^−^Na^+^ (95 mg, 0.23 mmol) in acetone (40.0 mL). The reaction mixture was stirred for 10 h. The solution was filtered and evaporated up to a minimum volume. The product was purified by column chromatography on silica gel (eluent: acetone, R_f_ = 0.5). Yield: 74 mg, (41.6%), m.p. 157–159 °C (decomp.), elem. anal. calc. (%) for C_24_H_27_Cl_6_N_5_NaO_2_RuS: C 36.66, H 3.46, N 8.91, and S 4.08. Found: C 36.86, H 3.62, N 8.59, and S 3.84, ESI-MS: *m*/*z*: 765 [M–Na^+^]^−^.

### 3.10. Na[trans-Ru(DMSO)(C_24_H_25_Cl_2_N_5_O)Cl_4_] *(**18**)*

Compound **12** (100 mg, 0.21 mmol) in ~3 mL of acetone was added to the solution of [Ru(DMSO)_2_Cl_4_]^−^Na^+^ (90 mg, 0.21 mmol) in acetone (40.0 mL). The reaction mixture was stirred for 10 h. The solution was filtered and evaporated up to a minimum volume. The product was purified by column chromatography on silica gel (eluent: acetone, R_f_ = 0.5). Yield: 113 mg (65.3%), m.p. 90–92°C (decomp.), elem. anal. calc. (%) for C_26_H_31_Cl_6_N_5_NaO_2_RuS*1C_3_H_6_O: C 39.92, H 4.27, N 8.03, and S 3.68. Found: C 40.07, H 4.36, N 7.76, and S 3.29, ESI-MS: *m*/*z*: 791 [M–Na^+^]^−^.

### 3.11. Na[trans-Ru(DMSO)(C_26_H_29_Cl_2_N_5_O)Cl_4_] *(**19**)*

Compound **13** (100 mg, 0.20 mmol) in ~3 mL of acetone was added to the solution of [Ru(DMSO)_2_Cl_4_]^−^Na^+^ (85 mg, 0.20 mmol) in acetone (40.0 mL). The reaction mixture was stirred for 10 h. The solution was filtered and evaporated up to a minimum volume. The product was purified by column chromatography on silica gel (eluent: acetone, R_f_ = 0.5). Yield: 121 mg (71.6%), m.p. 78–80 °C (decomp.), elem. anal. calc. (%) for C_28_H_35_Cl_6_N_5_NaO_2_RuS*0.5C_3_H_6_O: C 40.65, H 4.39, N 8.04, and S 3.68. Found: C 40.62, H 4.54, N 7.67, and S 3.43, ESI-MS: *m*/*z*: 819 [M–Na^+^]^−^.

### 3.12. Na[trans-Ru(DMSO)(C_30_H_37_Cl_2_N_5_O)Cl_4_] *(**20**)*

Compound **14** (70 mg, 0.13 mmol) in ~3 mL of acetone was added to the solution of [Ru(DMSO)_2_Cl_4_]^−^Na^+^ (53 mg, 0.13 mmol) in acetone (30.0 mL). The reaction mixture was stirred for 10 h. The solution was filtered and evaporated to a minimum volume. The product was purified by column chromatography on silica gel (eluent: acetone, R_f_ = 0.5). Yield: 70 mg (59.8%), m.p. 120–122 °C (decomp.), elem. anal. calc. (%) for C_32_H_43_Cl_6_N_5_NaO_2_RuS: C 42.77, H 4.82, N 7.79, and S 3.57. Found: C 43.04, H 5.23, N 8.01, and S 3.34, ESI-MS: *m*/*z*: 875 [M–Na^+^]^−^.

### 3.13. Electrochemical Activity

All electrochemical measurements were carried out under argon at room temperature. Cyclic voltammetry (CV) experiments were performed in KO264 PAR three-electrode microcells in CH3CN solution with 0.05 M of Bu_4_NBF_4_ as a supporting electrolyte using a IPC-Win potentiostat. The number of transferred electrons was determined by comparing to the height of the Fc^2+^/Fc^3+^ wave for the same concentration. A glassy carbon (GC) working electrode (diameter 2 mm), a platinum wire auxiliary electrode, and an aqueous Ag/AgCl/KCl (sat.) reference electrode was used. Solvents were routinely distilled and dried prior to use.

### 3.14. Stability

The stability of Ru(III) complexes was studied by electron absorption spectroscopy in 20 mM of phosphate buffer, pH 7.4, and 100 mM of NaCl. The working solution (2 mL, 200 μM complex) was prepared by diluting 10 μL of the original 40 mM solution in DMSO and 1.99 mL of phosphate buffer. UV-vis spectra were recorded every 60 s in the range of 280–600 nm at 37 °C. The half-transformation time was t_1/2_. For λ_max_, ΔA(t) was plotted, where ΔA = A_0_–A_i_, an initial section, was approximated as a linear function. At ΔA_line_./2 point calculated as t = t_1/2_.

### 3.15. Lipophilicity

*N*-octanol was saturated with water and with water saturated with n-octanol; mixture400 mL of n-octanol (water) and 100 mL of water (n-octanol) was stirred for 24 h; and then fractions were separated. The sample of the complex was dissolved in *n*-octanol and a series of solutions in octanol (300, 250, 3 × 200, 150, 100, and 50 μM) was prepared. Absorption spectra were recorded and the calibration curve for maximum absorption was plotted. From 200 μM solutions, the mixtures with water were prepared (1:1, 1:2, 2:1 *v*/*v*) and shaken for 15 min. The organic phase was separated by centrifugation. The concentration of the complex in *n*-octanol was determined from the calibration curve and lipophilicity (logP) was calculated as log *P* = log [(*C*_0_–*C*_aq_)/*C*_aq_].

### 3.16. Cellular Accumulation of Ruthenium Determined by Atomic Absorption Spectrometry

The intracellular accumulation of metal-containing compounds was determined as described [46,52,53]. The MCF-7 breast carcinoma cell line (CLS, Eppelheim, Germany) was propagated in Dulbecco’s modified Eagle medium supplemented with 10% foetal bovine serum (Biochrom GmbH, Germany) and 50 mg/mL gentamicin at 37 °C, as well ass 5% CO_2_ in a humidified atmosphere. Cells were grown until 80% confluence in 75 cm^2^ flasks. Stock solutions of **16** and **20** (10 mM in DMSO) were diluted with the full medium or serum-free medium immediately prior to cell exposure. Cells were treated with **16** or **20** (20 μM each) at 37 °C, 5% CO_2_, for up to 24 h, washed twice with PBS, and isolated by scraping off and centrifuged at 1000× *g* for 5 min.

For metal and protein quantification, pellets were resuspended in 250 µL of deionised water and lysed for 30 min with sonication. The protein content in lysates was determined by the Bradford method. For ruthenium measurements, a contrAA 700 high resolution continuum-source atomic absorption spectrometer (Analytik Jena AG, Jena, Germany) was used. Samples of the respective complex were used as standards. Calibration was done in a matrix-matched manner, that is, all samples and standards were adjusted to the same protein concentration of 1 mg/mL by dilution with water. Triton-X 100 (1%, 10 μL) and nitric acid (13%, 10 μL) were added to each standard sample (100 μL). Samples were injected (25 μL) into coated graphite tubes (Analytik Jena AG, Jena, Germany) and thermally processed as described with minor modifications [53]. Drying steps were adjusted, the atomisation temperature was set to 2400 °C, and the reading time was increased to 7 s. Ruthenium was quantified at 349.8945 nm. The mean integrated absorbance of triple injections was used throughout the measurements. Results (average of the two experiments) were expressed as nmol metal/mg protein.

### 3.17. Inhibition of TrxR

#### 3.17.1. Inhibition of Purified Protein

The activity of TrxR [40,54] was determined in a microplate format. Commercially available rat liver TrxR (Sigma Aldrich, St. Louis, MO, USA) was diluted with distilled water to 3.5 Unit/mL. In total, 25 μL aliquots of this solution were mixed with 25 μL of a potassium phosphate buffer, pH 7.0, with or without tested compounds. Fifty µL of 0.5% *v*/*v* dimethyl formamide in buffer was served as a blank. An additional control experiment revealed that the test compounds did not exhibit any absorption at the respective wavelength or reduce the DTNB in the absence of the enzyme. For this purpose, 25 µL of the highest test concentration of each compound and 25 µL of phosphate buffer (no enzyme) were mixed. Samples were incubated with moderate shaking for 75 min at 37 °C in a 96-well plate. To each well, 225 μL of the reaction mixture (500 μL of potassium phosphate buffer, pH 7.0, 80 μL of 100 mM ethylenediaminetetraacetic acid (EDTA), 20 μL of 0.2% bovine serum albumin, 100 μL of 20 mM NADPH (nicotinamide adenine dinucleotide phosphate), and 300 μL of water) was added; the reaction was initiated by the addition of 25 μL of 20 mM DTNB (5,5′-dithiobis(2-nitrobenzoic acid)) solution in ethanol. The formation of 5-TNB was monitored at 405 nm 10 times at 35 s intervals by a VICTOR X4 Plate Reader (Perkin Elmer). The increase of the 5-TNB concentration over time showed a linear trend (r^2^ ≥ 0.99); enzymatic activities were calculated as slopes (an increase of absorbance per second). IC_50_ values were calculated as the concentration of the compound that decreased the enzymatic activity of the control (no compound) by 50%. Values are mean ± SD of the three independent measurements.

#### 3.17.2. Inhibition of Intracellular TrxR1

The TrxR1 enzyme was measured in the extracts from the liver of male white outbred rats homogenised in 50 mM of PBS containing 1 mM of EDTA and centrifuged for 15 min at 10,000 rpm at 4 °C. The supernatant was adjusted to 7.5 µg/mL of protein as determined with pyrogallol red. Then, 40 μL of the enzyme solution was mixed with 10 μL of the test compounds in a 96-well clear flat bottom plate. After 10 min at 25 °C, 30 μL of PBS containing 0.7 mg/mL of BSA and 0.8 mM of NADPH was added. After 15 min at 25 °C, 20 µL of DTNB solution was added to the desired final concentrations. Kinetic studies were carried out by assaying TrxR1 at various concentrations of DTNB. The optical density of the samples was measured with an Infinite M200 Pro reader (Tecan, Grödig, Austria) at 412 nm every 30 s for 20 min. The activity of TrxR1 was calculated as a reaction slope relative to the control wells using Prism 8.0 (GraphPad, Inc., San Diego, CA, USA).

### 3.18. Cell Death Studies

The antiproliferative activity was studied by MTT assays as published previously [35]. For flow cytometry studies, cells were plated into 6-well plates (Eppendorf, Germany; 4 × 10^5^ cells in 2 mL of DMEM) and incubated for 24 h. Solutions of complexes in DMSO were prepared immediately prior to the day of the experiments. Cells were treated with either 20 µM of cisplatin, 50 µM of **16,** or 20 µM of **20**. Concentrations corresponded to two-fold IC_50_ values based on MTT assays. Cells were incubated for 24 h, pooled, washed with cold PBS, and resuspended in DMEM. Aliquots of cells were processed as recommended in the Muse Annexin V&Dead Cell Kit or Muse Caspase-3/7 Kit (Luminex). Measurements were carried out on a Muse Cell Analyser, Luminex corp., TX, USA.

### 3.19. In Vivo Acute Toxicity

The Balb/c female mice (8–10 weeks old, weight 20–22 g) were bred and hosted at the animal facility of the Blokhin Cancer Center [55]. Mice were kept at 21 ± 1 °C, 50–60% humidity; food and water were added ad libitum. All manipulations were performed in accordance to the European Convention for the Protection of Vertebrate Animals used for Experimental and other Scientific Purposes (ETS 123). Compound **16** was injected i.p.; 70–110 mg/kg single bolus administration in 200 µL of saline. Each cohort contained six mice. Animals were monitored for 21 days after injection. General behavioural activity, nutritional habits, and the integrity of hair cover were the criteria of acute toxicity.

## 4. Conclusions

Complexes in which the ruthenium fragment and lonidamine were connected by an imidazole linker were obtained and described. These complexes showed micromolar cytotoxicity and lipophilicity between 0.5 and 1.5. Complexes were more active than the corresponding ligands, the parent drug, and, in certain cases, cisplatin. The cytotoxicity increased along with the linker’s length. The most stable and biologically active complex was **20** and its half-transformation time was ~35 min. The intracellular accumulation of ruthenium complexes was fast and dependent on the length of the linker. The caspase 3/7 mediated apoptosis is a major mode of cell death induced by lonidamine-Ru complexes. Together with the tolerance of **16** in Balb/c mice, our data suggest a perspective of the new chemotype in search of antitumour drug candidates.

## Figures and Tables

**Table 1:** Half-transformation times and log P values for Ru(III) complexes.

Compound	**15**	**16**	**17**	**18**	**19**	**20**
Linker, *n*	2	3	4	6	8	12
t_1/2_, sec	360 ± 20	330 ± 20	360 ± 20	650 ± 30	1390 ± 70	2150 ± 110
Log P	*	*	*	0.6 ± 0.1	±0.08	1.3 ± 0.2

* complexes are unstable in aqueous solutions. Results are expressed as mean ± SD from three independent experiments.

**Table 2:** Redox potentials of complexes determined with a Pt and the glassy carbon (GC) working electrodes in CH_3_CN (C = 10^−3^ M, 0.5 M n-TBABF_4_, scan rate 100 mV/s, and Ag/AgCl (sat.)).

Complex	Pt			GC		
	E^ox^, V		E^red^	E^ox^, V		E^red^, V
	E_1_^ox^	E_2_^ox^		E_1_^ox^	E_2_^ox^	
**15**	1.290/1.185	1.850/1.630	-	1.270/1.17	1.890/1.650	-
**16**	1.300/1.190	1.830/1.630	−0.310/−0.090	1.280/1.195	-	−0.360/−0.190
**17**	1.290/1.190	1.820/1.610	−0.290/−0.080	1.300/1.196	1.870/1.640	−0.365/−0.128
**18**	1.280/1.170	1.820/1.620	−0.305/−0.095	1.310/1.192	1.860/1.600	−0.410/−0.150
**19**	1.320/1.190	1.840/1.620	−0.310/−0.105	1.308/1.200	1.950/1.620	−0.384/−0.135
**20**	-	-	-	1.498/1.137	1.873/1.609	−0.350/−0.190

**Table 3:** Redox potentials of complexes determined with a Pt and the glassy carbon (GC) working electrodes in CH_2_Cl_2_ (C = 10^−3^ M, 0.5 M n-TBABF_4_, scan rate 200 mV/s, and Ag/AgCl (sat.)).

Complex	Pt			GC		
	Eox, V		E red E1ox	Eox, V		E red
	E1ox	E2ox		E2ox	E1ox	
**15**	0.410/0.350	1.290/1.170	-	1.157/1.080		−0.290/−0.231
**16**	0.470/0.340	1.280/1.190	−0.540/−0.410	-		−0.490/−0.380
**17**	0.580/0.370	-	−0.650/−0.430	1.150/1.430		−0.690/−0.290
**18**	0.570/0.340	1.430/1.270	−0.420/−0.280	1.150/1.350		−0.590/−0.410
**19**	0.250/0.150	1.390/1.160	−0.550/−0.420	1.190/1.410		−0.600/−0.420
**20**	-	1.410/1.290	-	1.470/1,111		−0,680/−0,356

**Table 4:** Antiproliferative activity against human tumour cells.

Compound	Linker, n	IC_50_, µM				
		A549	MCF-7	SH-SY5Y	SW480	HaCaT
cisplatin	N/A	23 ± 6.5	>30	9.5 ± 0.1	21.7 ± 0.5	10 ± 5
lonidamine	N/A	>90	30 ± 10	>30	>90	3 ± 1
**9**	2	50 ± 16	35 ± 6	ND	48 ± 10	23 ± 2
**10**	3	>90	>90	ND	>90	39 ± 4
**11**	4	25 ± 7	22 ± 6	ND	28 ± 8	45 ± 3
**12**	6	24 ± 10	17 ± 2.5	ND	16 ± 6	40 ± 4
**13**	8	15 ± 6	10 ± 2	ND	>90	45 ± 2
**14**	12	15.0± 2.2	20.8± 4.7	ND	8.4 ± 1.6	ND
**15**	2	>30	20.4 ± 0.1	25.8 ± 4.5	21.4 ± 1.8	ND
**16**	3	>30	23.0 ± 0.5	26.1 ± 5.5	25 ± 7.1	ND
**17**	4	>30	>30	27.7 ± 3.3	21 ± 1.8	ND
**18**	6	12.9 ± 1.0	22.0 ± 0.7	5.1 ± 2.5	19.6 ± 3.9	ND
**19**	8	5.9 ± 2.6	17.06 ± 0.3	2.64 ± 1.5	9.3 ± 0.1	ND
**20**	12	8.1 ± 1.1	9.2 ± 0.1	6.1 ± 0.3	9.6 ± 1.3	ND

Results of MTT assays after a 72 h cell exposure. Shown are mean ± SD from three independent experiments. ND, not determined.

**Table 5:** Drug-induced lethality in Balb/c mice injected with complex **16**.

Single Dose, mg/kg i.p.				
70	80	90	100	110
0/6 *	0/6	2/6	2/6	4/6

* died/survived (n = 6).

## Supplementary Materials

The following are available online at https://www.mdpi.com/article/10.3390/ijms222413468/s1.

## References

[1] Rademaker-Lakhai J.M., van den Bongard D., Pluim D., Beijnen J.H., Schellens J.H. (2004). A Phase I and pharmacological study with imidazolium-trans-DMSO-imidazole-tetrachlororuthenate, a novel ruthenium anticancer agent. Clin. Cancer Res..

[2] Jakupec M.A., Arion V.B., Kapitza S., Reisner E., Eichinger A., Pongratz M., Marian B., Graf von Keyserlingk N., Keppler B.K. (2005). KP1019 (FFC14A) from bench to bedside: Preclinical and early clinical development—An overview. Int. J. Clin. Pharmacol. Ther..

[3] Hartinger C.G., Jakupec M.A., Zorbas-Seifried S., Groessl M., Egger A., Berger W., Zorbas H., Dyson P.J., Keppler B.K. (2008). KP1019, A New Redox-Active Anticancer Agent—Preclinical Development and Results of a Clinical Phase I Study in Tumor Patients. Chem. Biodivers..

[4] Lentz F., Drescher A., Lindauer A., Henke M., Hilger R.A., Hartinger C.G., Scheulen M.E., Dittrich C., Keppler B.K., Jaehde U. (2009). Pharmacokinetics of a novel anticancer ruthenium complex (KP1019, FFC14A) in a phase I dose-escalation study. Anticancer Drugs.

[5] Alessio E. (2017). Thirty Years of the Drug Candidate NAMI-A and the Myths in the Field of Ruthenium Anticancer Compounds: A Personal Perspective. Eur. J. Inorg. Chem..

[6] Alessio E., Messori L. (2019). NAMI-A and KP1019/1339, Two Iconic Ruthenium Anticancer Drug Candidates Face-to-Face: A Case Story in Medicinal Inorganic Chemistry. Molecules.

[7] Coverdale J.P.C., Laroiya-McCarron T., Romero-Canelón I. (2019). Designing Ruthenium Anticancer Drugs: What Have We Learnt from the Key Drug Candidates?. Inorganics.

[8] Sava G., Gagliardi R., Bergamo A., Alessio E., Mestroni G. (1999). Treatment of metastases of solid mouse tumours by NAMI-A: Comparison with cisplatin, cyclophosphamide and dacarbazine. Anticancer Res..

[9] Vadori M., Florio C., Groppo B., Cocchietto M., Pacor S., Zorzet S., Candussio L., Sava G. (2015). The antimetastatic drug NAMI-A potentiates the phenylephrine-induced contraction of aortic smooth muscle cells and induces a transient increase in systolic blood pressure. J. Biol. Inorg. Chem..

[10] Brescacin L., Masi A., Sava G., Bergamo A. (2015). Effects of the ruthenium-based drug NAMI-A on the roles played by TGF-β1 in the metastatic process. J. Biol. Inorg. Chem..

[11] Bergamo A., Dyson P.J., Sava G. (2018). The mechanism of tumour cell death by metal-based anticancer drugs is not only a matter of DNA interactions. Coord. Chem. Rev..

[12] Hartinger C.G., Zorbas-Seifried S., Jakupec M.A., Kynast B., Zorbas H., Keppler B.K. (2006). From bench to bedside—Preclinical and early clinical development of the anticancer agent indazolium trans-[tetrachlorobis(1H-indazole)ruthenate(III)] (KP1019 or FFC14A). J. Inorg. Biochem..

[13] Burris H.A., Bakewell S., Bendell J.C., Infante J., Jones S.F., Spigel D.R., Weiss G.J., Ramanathan R.K., Ogden A., Von Hoff D. (2016). Safety and activity of IT-139, a ruthenium-based compound, in patients with advanced solid tumours: A first-in-human, open-label, dose-escalation phase I study with expansion cohort. ESMO Open.

[14] Bakewell S., Conde I., Fallah Y., McCoy M., Jin L., Shajahan-Haq A.N. (2020). Inhibition of DNA Repair Pathways and Induction of ROS Are Potential Mechanisms of Action of the Small Molecule Inhibitor BOLD-100 in Breast Cancer. Cancers.

[15] Neuditschko B., Legin A.A., Baier D., Schintlmeister A., Reipert S., Wagner M., Keppler B.K., Berger W., Meier-Menches S.M., Gerner C. (2021). Interaction with Ribosomal Proteins Accompanies Stress Induction of the Anticancer Metallodrug BOLD-100/KP1339 in the Endoplasmic Reticulum. Angew. Chem. Int. Ed..

[16] Warburg O. (1956). On the Origin of Cancer Cells. Science.

[17] Vander Heiden M.G., Cantley L.C., Thompson C.B. (2009). Understanding the Warburg Effect: The Metabolic Requirements of Cell Proliferation. Science.

[18] Pelicano H., Martin D.S., Xu R.H., Huang P. (2006). Glycolysis inhibition for anticancer treatment. Oncogene.

[19] Lis P., Dyląg M., Niedźwiecka K., Ko Y.H., Pedersen P.L., Goffeau A., Ułaszewski S. (2016). The HK2 Dependent “Warburg Effect” and Mitochondrial Oxidative Phosphorylation in Cancer: Targets for Effective Therapy with 3-Bromopyruvate. Molecules.

[20] Floridi A., Paggi M.G., D’Atri S., De Martino C., Marcante M.L., Silvestrini B., Caputo A. (1981). Effect of Lonidamine on the Energy Metabolism of Ehrlich Ascites Tumor Cells. Cancer Res..

[21] Nath K., Guo L., Nancolas B., Nelson D.S., Shestov A.A., Lee S.-C., Roman J., Zhou R., Leeper D.B., Halestrap A.P. (2016). Mechanism of antineoplastic activity of lonidamine. Biochim. Biophys. Acta BBA Rev. Cancer.

[22] Berruti A., Bitossi R., Gorzegno G., Bottini A., Alquati P., De Matteis A., Nuzzo F., Giardina G., Danese S., De Lena M. (2002). Time to progression in metastatic breast cancer patients treated with epirubicin is not improved by the addition of either cisplatin or lonidamine: Final results of a phase III study with a factorial design. J. Clin. Oncol..

[23] Armarego W.L.F., Chai C. (2003). Purification of Laboratory Chemicals.

[24] Cervantes-Madrid D., Romero Y., Dueñas-González A. (2015). Reviving Lonidamine and 6-Diazo-5-oxo-L-norleucine to Be Used in Combination for Metabolic Cancer Therapy. BioMed Res. Int..

[25] Gelemanović A., Vidović T., Stepanić V., Trajković K. (2021). Identification of 37 Heterogeneous Drug Candidates for Treatment of COVID-19 via a Rational Transcriptomics-Based Drug Repurposing Approach. Pharmaceuticals.

[26] Gil-Moles M., Türck S., Basu U., Pettenuzzo A., Bhattacharya S., Rajan A., Ma X., Büssing R., Wölker J., Burmeister H. (2021). Metallodrug Profiling against SARS-CoV-2 Target Proteins Identifies Highly Potent Inhibitors of the S/ACE2 interaction and the Papain-like Protease PLpro. Chem. Eur. J..

[27] Kenny R.G., Marmion C.J. (2019). Toward Multi-Targeted Platinum and Ruthenium Drugs—A New Paradigm in Cancer Drug Treatment Regimens?. Chem. Rev..

[28] Kostrhunova H., Zajac J., Markova L., Brabec V., Kasparkova J. (2020). A Multi-action PtIV Conjugate with Oleate and Cinnamate Ligands Targets Human Epithelial Growth Factor Receptor HER2 in Aggressive Breast Cancer Cells. Angew. Chem. Int. Ed..

[29] Tremlett W.D.J., Goodman D.M., Steel T.R., Kumar S., Wieczorek-Błauż A., Walsh F.P., Sullivan M.P., Hanif M., Hartinger C.G. (2021). Design concepts of half-sandwich organoruthenium anticancer agents based on bidentate bioactive ligands. Coord. Chem. Rev..

[30] Xu Z., Wang Z., Deng Z., Zhu G. (2021). Recent advances in the synthesis, stability, and activation of platinum(IV) anticancer prodrugs. Coord. Chem. Rev..

[31] Nazarov A.A., Gardini D., Baquie M., Juillerat-Jeanneret L., Serkova T.P., Shevtsova E.P., Scopelliti R., Dyson P.J. (2013). Organometallic anticancer agents that interfere with cellular energy processes: A subtle approach to inducing cancer cell death. Dalton Trans..

[32] Nosova Y.N., Foteeva L.S., Zenin I.V., Fetisov T.I., Kirsanov K.I., Yakubovskaya M.G., Antonenko T.A., Tafeenko V.A., Aslanov L.A., Lobas A.A. (2017). Enhancing the Cytotoxic Activity of Anticancer PtIV Complexes by Introduction of Lonidamine as an Axial Ligand. Eur. J. Inorg. Chem..

[33] Okulova Y.N., Zenin I.V., Shutkov I.A., Kirsanov K.I., Kovaleva O.N., Lesovaya E.A., Fetisov T.I., Milaeva E.R., Nazarov A.A. (2019). Antiproliferative activity of Pt(IV) complexes with lonidamine and bexarotene ligands attached via succinate-ethylenediamine linker. Inorg. Chim. Acta.

[34] Shutkov I.A., Antonets A.A., Tyurin V.Y., Milaeva E.R., Nazarov A.A. (2021). Ruthenium(III) Complexes of NAMI-A Type with Ligands Based on Lonidamine and Bexarotene as Antiproliferative Agents. Russ. J. Inorg. Chem..

[35] Nosova Y.N., Zenin I.V., Maximova V.P., Zhidkova E.M., Kirsanov K.I., Lesovaya E.A., Lobas A.A., Gorshkov M.V., Kovaleva O.N., Milaeva E.R. (2017). Influence of the Number of Axial Bexarotene Ligands on the Cytotoxicity of Pt(IV) Analogs of Oxaliplatin. Bioinorg. Chem. Appl..

[36] Holmgren A., Lu J. (2010). Thioredoxin and thioredoxin reductase: Current research with special reference to human disease. Biochem. Biophys. Res. Commun..

[37] Saccoccia F., Angelucci F., Boumis G., Carotti D., Desiato G., Miele A.E., Bellelli A. (2014). Thioredoxin reductase and its inhibitors. Curr. Protein Pept. Sci..

[38] Zhang B., Zhang J., Peng S., Liu R., Li X., Hou Y., Han X., Fang J. (2017). Thioredoxin reductase inhibitors: A patent review. Expert Opin. Ther. Pat..

[39] Zhang B., Liu Y., Li X., Xu J., Fang J. (2018). Small Molecules to Target the Selenoprotein Thioredoxin Reductase. Chem. Asian J..

[40] Shpakovsky D.B., Shtil A.A., Kharitonashvili E.V., Tyurin V.Y., Antonenko T.A., Nazarov A.A., Osipova V.P., Berberova N.T., Foteeva L.S., Schmidt C. (2018). The antioxidant 2,6-di-tert-butylphenol moiety attenuates the pro-oxidant properties of the auranofin analogue. Metallomics.

[41] Alessio E., Balducci G., Lutman A., Mestroni G., Calligaris M., Attia W.M. (1993). Synthesis and characterization of two new classes of ruthenium(III)-sulfoxide complexes with nitrogen donor ligands (L): Na[trans-RuCl_4_(R_2_SO)(L)] and mer, cis-RuCl_3_(R_2_SO)(R_2_SO)(L). The crystal structure of Na[trans-RuCl_4_(DMSO)(NH_3_)] 2DMSO, Na[trans-RuCl_4_(DMSO)(Im)] H_2_O, Me_2_CO (Im = imidazole) and mer, cis-RuCl_3_(DMSO)(DMSO)(NH_3_). Inorg. Chim. Acta.

[42] Schluga P., Hartinger C.G., Egger A., Reisner E., Galanski M., Jakupec M.A., Keppler B.K. (2006). Redox behavior of tumor-inhibiting ruthenium(III) complexes and effects of physiological reductants on their binding to GMP. Dalton Trans..

[43] Reisner E., Arion V.B., Guedes da Silva M.F.C., Lichtenecker R., Eichinger A., Keppler B.K., Kukushkin V.Y., Pombeiro A.J.L. (2004). Tuning of Redox Potentials for the Design of Ruthenium Anticancer Drugs—An Electrochemical Study of [trans-RuCl_4_L(DMSO)]- and [trans-RuCl_4_L_2_]- Complexes, where L = Imidazole, 1,2,4-Triazole, Indazole. Inorg. Chem..

[44] Ravera M., Baracco S., Cassino C., Zanello P., Osella D. (2004). Appraisal of the redox behaviour of the antimetastatic ruthenium(III) complex [ImH][RuCl_4_(DMSO)(Im)], NAMI-A. Dalton Trans..

[45] Reisner E., Arion V.B., Keppler B.K., Pombeiro A.J.L. (2008). Electron-transfer activated metal-based anticancer drugs. Inorg. Chim. Acta.

[46] Oehninger L., Stefanopoulou M., Alborzinia H., Schur J., Ludewig S., Namikawa K., Muñoz-Castro A., Köster R.W., Baumann K., Wölfl S. (2013). Evaluation of arene ruthenium(II) N-heterocyclic carbene complexes as organometallics interacting with thiol and selenol containing biomolecules. Dalton Trans..

[47] Bakulina O., Bannykh A., Jovanović M., Domračeva I., Podolski-Renić A., Žalubovskis R., Pešić M., Dar’in D., Krasavin M. (2019). Design, synthesis, and biological evaluation of novel derivatives of dithiodiglycolic acid prepared via oxidative coupling of thiols. J. Enzym. Inhib. Med. Chem..

[48] Omata Y., Folan M., Shaw M., Messer R.L., Lockwood P.E., Hobbs D., Bouillaguet S., Sano H., Lewis J.B., Wataha J.C. (2006). Sublethal concentrations of diverse gold compounds inhibit mammalian cytosolic thioredoxin reductase (TrxR1). Toxicol. In Vitro.

[49] Hashemy S.I., Ungerstedt J.S., Avval F.Z., Holmgren A. (2006). Motexafin Gadolinium, a Tumor-selective Drug Targeting Thioredoxin Reductase and Ribonucleotide Reductase. J. Biol. Chem..

[50] Citta A., Folda A., Scutari G., Cesaro L., Bindoli A., Rigobello M.P. (2012). Inhibition of thioredoxin reductase by lanthanum chloride. J. Inorg. Biochem..

[51] Vermes I., Haanen C., Steffens-Nakken H., Reutellingsperger C. (1995). A novel assay for apoptosis Flow cytometric detection of phosphatidylserine expression on early apoptotic cells using fluorescein labelled Annexin V. J. Immunol. Methods.

[52] Schatzschneider U., Niesel J., Ott I., Gust R., Alborzinia H., Wölfl S. (2008). Cellular Uptake, Cytotoxicity, and Metabolic Profiling of Human Cancer Cells Treated with Ruthenium(II) Polypyridyl Complexes [Ru(bpy)_2_(N N)]Cl_2_ with NN = bpy, phen, dpq, dppz, and dppn. ChemMedChem.

[53] Appold M., Mari C., Lederle C., Elbert J., Schmidt C., Ott I., Stühn B., Gasser G., Gallei M. (2017). Multi-stimuli responsive block copolymers as a smart release platform for a polypyridyl ruthenium complex. Polym. Chem..

[54] Schmidt C., Karge B., Misgeld R., Prokop A., Franke R., Brönstrup M., Ott I. (2017). Gold(I) NHC Complexes: Antiproliferative Activity, Cellular Uptake, Inhibition of Mammalian and Bacterial Thioredoxin Reductases, and Gram-Positive Directed Antibacterial Effects. Chem. Eur. J..

[55] Grin A.M., Tikhonov I.S., Petrova S.A., Pogorilyy A.V., Noev N.A., Tatarskiy V.V., Shpakovsky B.D., Milaeva R.E., Kalinina V.E., Chernov N.N. (2020). New Derivatives of Bacteriopurpurin with Thiolated Au (I) Complexes: Dual Darkand Light Activated Antitumor Potency. Anti-Cancer Agents Med. Chem..

