# Supplementary material for: Ru(III) Complexes with Lonidamine-Modified Ligands
Source: Int J Mol Sci. 2021 Dec 15;22(24):13468. doi: 10.3390/ijms222413468 (PMC8707700; doi:10.3390/ijms222413468)
Supplement: Supplementary file 1 [file ijms-22-13468-s001.zip › ijms-1511111-supplementary.pdf]

Supplementary Information

Ru(III) COMPLEXES WITH LONIDAMINE MODIFIED LIGANDS

Ilya A. Shutkov, Yulia N. Okulova, Vladimir Yu. Turin, Elena V. Sokolova, Denis A. Babkov, Alexander A. Spasov, Yulia A. Gracheva, Claudia Schmidt, Kirill I. Kirsanov, Alexander A. Shtil, Olga M. Redkozubova, Elena F. Shevtsova, Elena R. Milaeva, Ingo Ott and Alexey A. Nazarov *

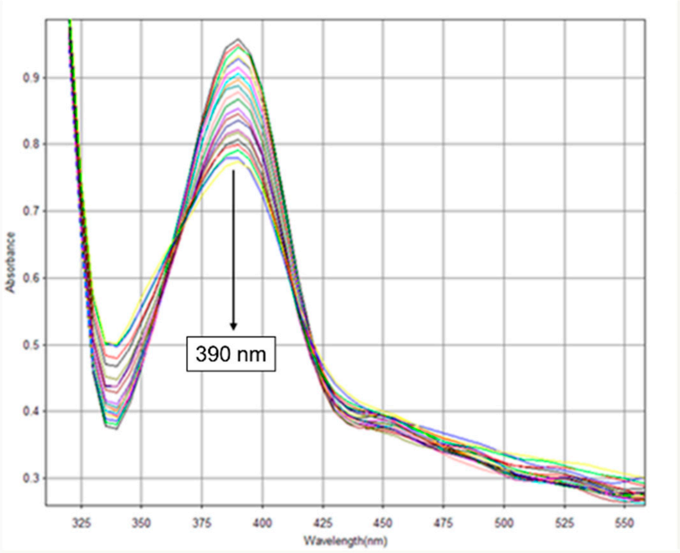

Figure S1. Electronic absorption spectra of complex **16**.

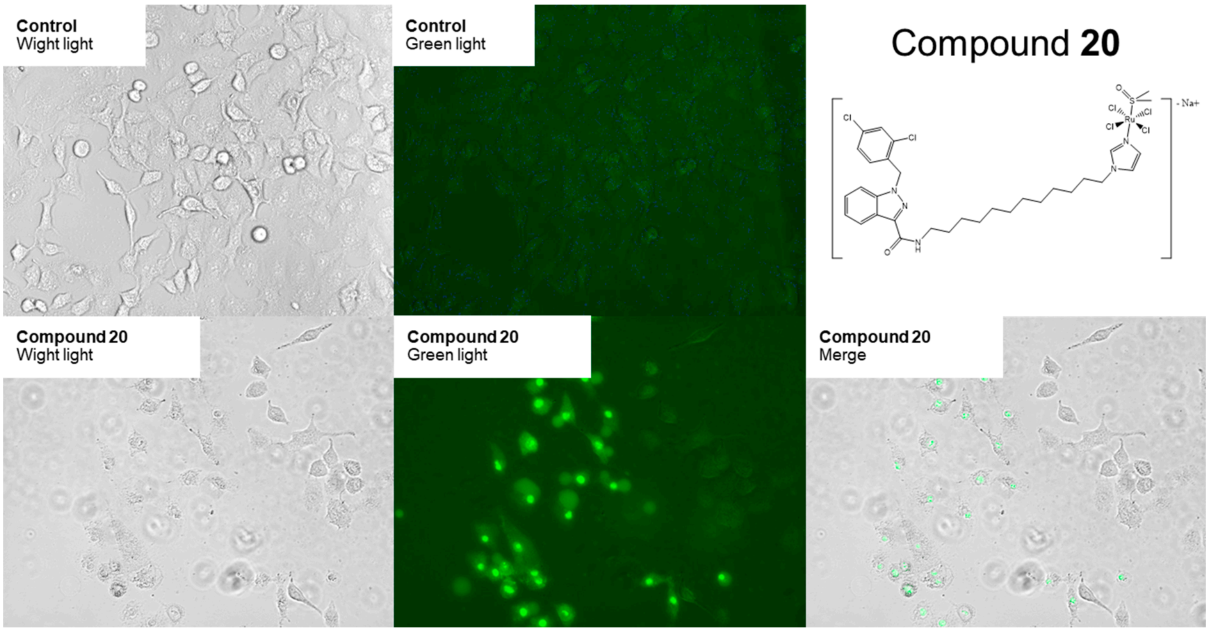

Figure S2. Complex **20** in caspase activation fluorescent kit CellEvent™ Caspase-3/7 Green ReadyProbes.

Table S1. Inhibition of TrxR1 in rat liver extracts.

DTNB, mM	TrxR1 IC_{50}, µM (95% C.I.)	
	**16**	**20**
2.5	33 (19−56)	15 (6−36)
0.1	33 (18−60)	15 (4−44)
0.06	24 (8−69)	17 (8−27)
